# Bringing fear into focus: The intersections of HIV and masculine gender norms in Côte d’Ivoire

**DOI:** 10.1371/journal.pone.0223414

**Published:** 2019-10-23

**Authors:** Danielle Amani Naugle, Natalie Jean Tibbels, Zoé Mistrale Hendrickson, Abdul Dosso, Lynn Van Lith, Elizabeth C. Mallalieu, Anne Marie Kouadio, Walter Kra, Diarra Kamara, Patricia Dailly-Ajavon, Adama Cissé, Kim Seifert-Ahanda, Sereen Thaddeus, Stella Babalola, Christopher J. Hoffmann

**Affiliations:** 1 Johns Hopkins Center for Communication Programs, Johns Hopkins Bloomberg School of Public Health, Baltimore, Maryland, United States of America; 2 Johns Hopkins Center for Communication Programs, Johns Hopkins Bloomberg School of Public Health, Abidjan, Côte d’Ivoire; 3 Sociology Department, Félix Houphouët-Boigny University in Cocody, Abidjan, Côte d’Ivoire; 4 Sociology Department, Alassane Ouattra University, Bouaké, Côte d’Ivoire; 5 United States Agency for International Development, Washington, DC, United States of America; 6 School of Medicine, Johns Hopkins University, Baltimore, Maryland, United States of America; University of South Florida, UNITED STATES

## Abstract

This qualitative research study explored the role of masculinity in men’s engagement in the HIV care continuum in Côte d’Ivoire. The researchers conducted 73 in-depth interviews and 28 focus group discussions with 227 Ivoirian men between November and December 2016 across three urban sites. Participants in the study expressed that fear was the primary barrier to HIV testing and treatment. These men described five value domains–health, sexuality, work and financial success, family, and social status. Men saw HIV as a direct threat to their agency and strength with respect to each of these value domains, thus shedding light on their reluctance to discover their HIV status through HIV testing. With this data, the researchers created the Masculine Values Framework, a descriptive framework of masculine values that can be applied to better understand the behavior men exhibit in Côte d’Ivoire in the face of HIV. The Masculine Values Framework offers practical guidance for developing gender-sensitive HIV-focused social and behavior change programming in Côte d’Ivoire and similar contexts to reach the UNAIDS 90-90-90 targets.

## Introduction

The global burden of HIV disproportionately affects sub-Saharan Africa [1]. Antiretroviral therapy (ART) and targeted prevention strategies can substantially reduce HIV incidence and HIV-related mortality [2]. To accelerate the global reduction in HIV incidence and HIV-related mortality, the Joint United Nations Programme on HIV/AIDS (UNAIDS) established the ambitious 90-90-90 targets: by 2020, 90% of people living with HIV will know their HIV status, 90% of people diagnosed with HIV will receive sustained ART, and 90% of people receiving ART will achieve viral suppression [3].

In Côte d’Ivoire, an estimated 2.5% of adults of reproductive age (15–49) are living with HIV [4]. Despite relatively high HIV awareness and widely available free HIV services (including medications) in Côte d’Ivoire, HIV testing and treatment uptake are inadequate to reach the 90-90-90 goals by 2020, especially among men. Only 24.2% of men living with HIV (MLHIV) know their HIV status; 70.7% of the MLHIV who know their HIV status self-report current use of ART; and 65.2% of men who self-report current use of ART are virally suppressed (compared to 43.2%, 92.6%, and 77.9% of women living with HIV, respectively) [4].

In Côte d’Ivoire, as in other countries, men engage in care less than women at all points along the HIV care continuum [5, 6, 7, 8, 9, 10, 11, 12, 13, 14, 15]. Research from across the African continent suggests that prevailing narratives of masculinity are often at odds with perceptions of disease and health-care seeking [16, 17, 18]. Assimilating these narratives of masculinity can have long-term tangible effects on men’s health outcomes [19, 20, 21, 22, 23]. For example, concepts of *strength* and *resilience* may prevent men from engaging in timely HIV testing and care if they view health care seeking as an outward sign of diminished physical strength [24, 25, 26, 27, 28, 29, 30, 31, 32]. In addition, the possibility of an HIV-positive diagnosis may threaten a man’s identity as a lover, husband, father, or provider, thereby leading him to avoid HIV testing [31, 32, 33, 34, 35, 36, 37, 38, 39, 40, 41, 42, 43].

To deepen our understanding of the reluctance men exhibit around HIV testing in Côte d’Ivoire, we sought to understand the aspirations and values of Ivoirian men through the lens of masculinity. We then explored how an HIV-positive diagnosis potentially threatens men’s aspirations, creating obstacles to HIV testing. We drew on men’s voices to inform a conceptual framework of the intersections between men’s values and HIV testing.

## Methods

The study was conducted in three urban sites in Côte d’Ivoire: Abidjan (Yopougon-Ouest-Songon), Bouaké, and San Pédro. The sites were chosen due to the higher-than-national prevalence of HIV among men and women of reproductive age. Based on the 2011–2012 Demographic Health Survey (DHS), HIV prevalence among men aged 15 to 49 in Côte d’Ivoire was 2.9% nationally, and 4.1% in Abdijan, 2.2% in the Centre-Nord region, where Bouaké is located, and 3.4% in the Sud-Ouest region, where San Pédro is located [44]. HIV prevalence among women aged 15 to 49 in Côte d’Ivoire was 4.7% nationally, and 5.9% in Abijdan, 6.3% in the Centre-Nord region, and 5.2% in the Sud-Ouest region [44].

In-depth interviews (IDIs) and focus group discussions (FGDs) were conducted with participants to understand their aspirations and values and to explore how they perceived health care seeking, illness, and HIV. Purposive sampling was used to recruit men between 25 and 49 years of age working in agricultural, industrial, public/private, and employed/unemployed sectors. Participants fell into one of three categories: (a) men whose HIV status was unknown to investigators, (b) men who were living with HIV (MLHIV) and in treatment, and (c) MLHIV and not in treatment. Participants were recruited in-person using recruitment scripts for either one IDI or FGD with the help of local HIV-focused non-governmental organizations (NGOs). The study team contacted men who expressed interest in participating in the study to arrange a time and a place for the IDI or FGD.

MLHIV were recruited for one IDI; men of unknown HIV status were recruited for either one IDI (to understand individual-level views) or one FGD (to understand normative views). Men whose HIV-status was unknown to the investigators may have tested in the past, but were not asked to disclose the results of prior HIV testing during the IDI or FGD. IDIs and FGDs were conducted in settings convenient for participants and where interviewers and facilitators were able to ensure participants’ privacy—most often in NGO offices or conference rooms. Interviewers and facilitators were male Ivoirian researchers with doctorates in sociology and experience in qualitative data collection. They were trained in the study protocol and research ethics by author DN. Alongside other data collectors, author WK conducted interviews. Researchers and participants had no prior relationship. Written informed consent was obtained from all individual participants prior to data collection. Researchers used pre-tested, structured guides to facilitate conversations. The data collection team conducted daily debriefs to organize field notes, identify themes emerging from the IDIs and FGDs, and discuss data saturation. Those themes were organized into a field report. IDIs and FGDs were conducted in French, audio-recorded, transcribed, and analyzed in French. Transcripts were not returned to participants for comment. (More information is provided in the S1 COREQ checklist.) The study was reviewed and approved by the Johns Hopkins Bloomberg School of Public Health Institutional Review Board (IRB#00007374) and the National Ethics Research Committee (Comité National d’Ethique de la Recherche) in Côte d’Ivoire (097/MSLS/CNER-dk).

We developed a structured coding framework using both deductive and inductive approaches and iteratively refined them by reading transcripts. Deductive codes were defined prior to analysis and were informed by the extant research on gender and masculinity [24, 45] as well as by behavioral theory [46, 47]. Inductive codes emerged from an initial reading of a subset of transcripts. After open coding took place, codes reflecting similar themes were grouped together and/or merged and new codes or sub-codes were incorporated into the master codebook. Twenty percent of all transcripts were double coded in Atlas.ti by two of five coders; discrepancies were resolved by consensus and/or consultation with a third coder.

Through an iterative approach, rounds of coding were followed by participatory data analysis sessions that facilitated hypothesis generation, analysis of emergent themes, and coding and analysis of additional transcripts. Once the coding was complete, the sections of transcript corresponding to each code were extracted and reviewed by multiple members of the coding team to corroborate themes identified during coding and to identify additional themes. We then analyzed the themes through participatory data analysis sessions and discussed data saturation. Findings were cross-checked with the field report and shared with local stakeholders and experts through a research dissemination workshop. Participants did not attend this workshop due to the sensitive nature of the study. In the following section, we present findings from thematic analyses that illustrate the intersections between male aspirations and values, masculinity, and HIV. Quotations have been translated into English for this manuscript.

## Results

We conducted 73 IDIs and 28 FGDs with a total of 227 Ivoirian men between November and December 2016. Four men declined to participate in the study after being administered informed consent. The IDIs lasted about 45 minutes, and the FGDs lasted about an hour and 15 minutes. Table 1 shows the number of participants by data collection method, geographic area, age group, and HIV status.

**Table 1:** Participant characteristics.

**Participant Characteristics**	**Percent**	**N**
Total Participants	100%	227
In-depth interviews	32%	73
Focus group discussions (n = 28)	68%	154
Geographic area		
Abidjan	35%	80
Bouaké	33%	74
San Pédro	32%	73
Age group		
25–34	44%	100
35–49	56%	127
HIV status		
Unknown to investigators	88%	199
MLHIV, in treatment	7%	15
MLHIV, not in treatment	6%	13
**Participant Characteristics**	**N**	**Percent**
Total Participants	227	100%
In-depth interviews	73	32%
Focus group discussions (n = 28)	154	68%
Geographic area		
Abidjan	80	35%
Bouaké	74	33%
San Pédro	73	32%
Age group		
25–34	100	44%
35–49	127	56%
HIV status		
Unknown to investigators	199	88%
MLHIV, in treatment	15	7%
MLHIV, not in treatment	13	6%

Data collection took place before the roll-out of Test and Treat in early 2018, which eliminated national eligibility criteria for accessing free antiretroviral treatment. Most of the MLHIV who participated in the study and were not currently in treatment self-reported being ineligible for treatment based on criteria that were in effect at the time (a CD4 count <500 cells/mm^3^ or the presence of active tuberculosis disease). However, study participants whose HIV status was unknown to investigators did not spontaneously cite treatment eligibility criteria as a barrier to HIV testing or treatment.

In this study of the role of masculinity in men’s engagement in the HIV care continuum in Côte d’Ivoire, we identified five core value domains: health, sexuality, work and financial success, family, and social status. In general, all of the men in our study upheld these domains irrespective of their geographic location, age, HIV, or treatment status; however, health was more salient to HIV-positive men than to men of unknown status and younger men placed greater emphasis on work and sexuality. We also identified primary barriers to HIV testing and determined that fear was chief among them. This fear was at times attributed to an association between HIV, physical illness, and death. Outdated perceptions of HIV treatment accessibility and effectiveness led men to view an HIV-positive diagnosis as a death sentence, both physically and socially.

Even more than a fear of illness and death, men expressed fear of the impact of HIV on non-health-related aspirations. Some men expressed preferring to die of HIV without ever knowing their status than to be diagnosed HIV-positive. One man explained that his reluctance to test was due to his fear of living with an HIV-positive diagnosis, not his fear of death. He explained: “Frankly, I said no, I can’t, because I do not have the courage. Because, if the doctor ever did my test and gave me a positive result, I could not bear it. So I prefer to stay like that rather than to get tested and be told that I have AIDS” (FGD, man of unknown status, 25–34, Bouaké).

To understand men’s reluctance to get tested for HIV, we explored their aspirations and the relationships between those aspirations and HIV. In general, with the exception of MLHIV, participants did not mention health as an aspiration unprompted; but, when occasioned, many men expressed that good health was necessary to attain or maintain their other values. These values were linked and mutually reinforcing. We did not observe geographic, age, or HIV- or treatment status-related differences in terms of men’s overall values, but the relative importance of each value did vary across individual participants.

### Masculine constructs of agency and strength

Within each of the five value domains–health, sexuality, work and financial success, family, and social status–we identified constructs of agency and strength. For purposes of this study, *agency* denotes the capacity to choose and to act, to make independent decisions, to be in control, and not to depend on others for decision-making, direction, food or shelter [48]. We used *strength* to reflect physical, psychological and economic strength, power, pride, and invulnerability. Many participants referred to strength in French as *force* or *pouvoir*.

**Fig 1:**
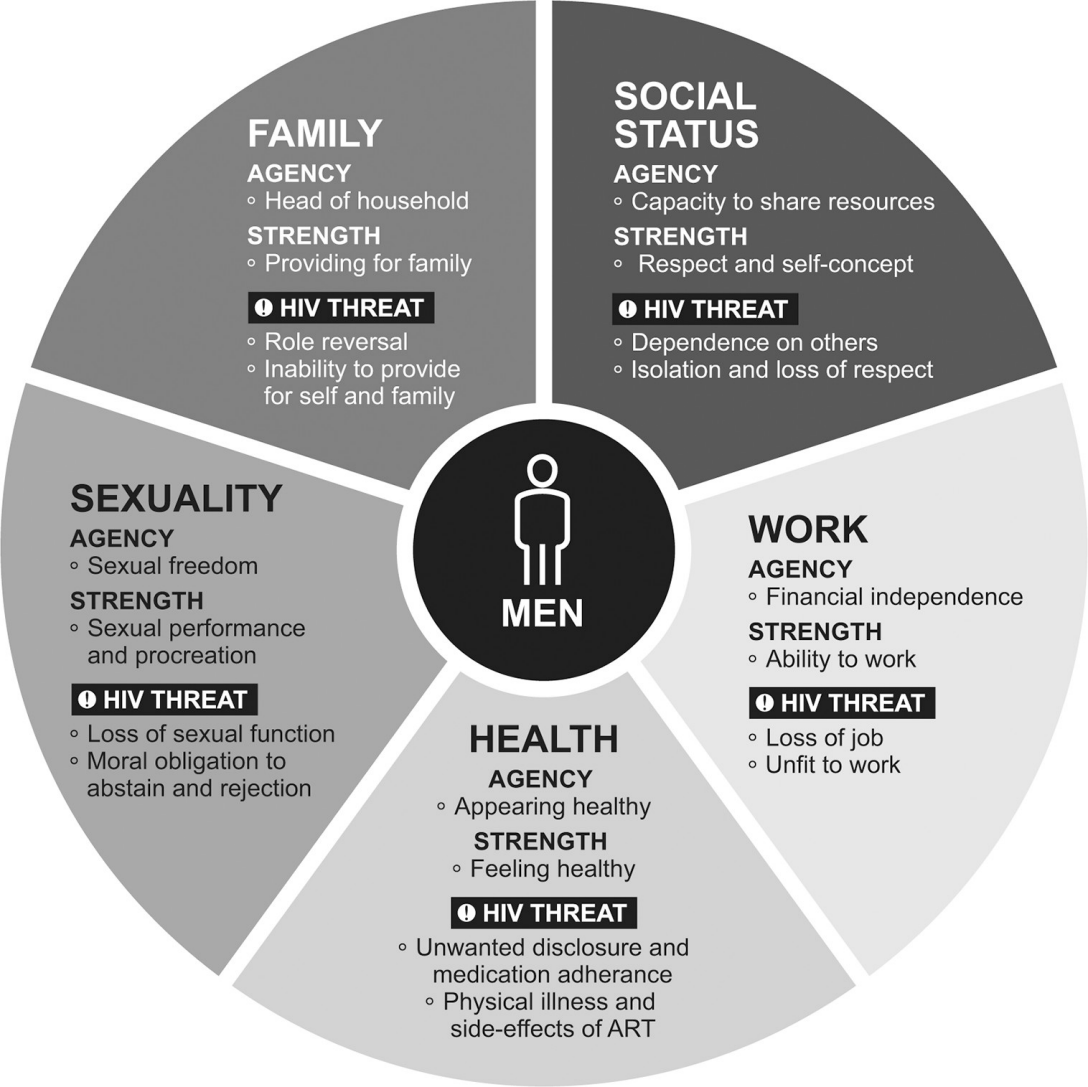
Masculine Values Framework and the threat of HIV among Ivoirian men. The Masculine Values Framework (MVF) outlines the constructs of agency and strength with respect to each of the five core values described by the Ivoirian men in the study–health, sexuality, family, social status and work–and the threat HIV posed to each value.

Much of the fear men expressed regarding an HIV-positive diagnosis reflected anxiety about losing agency and strength with respect to each value domain. We schematically represent these relationships in the Masculine Values Framework (MVF); situating health as the foundation for sexuality, work, family, and social status because it was viewed as necessary to attain the others (Fig 1). With respect to each value, we list the potential threat of HIV. Each of the following sections describes the value domains overall and in terms of agency and strength.

#### Health

Participants described health as foundational and necessary to achieving the other values. Without health, a man might not have a job, a family, an active sex life, or social status. A MLHIV in treatment in San Pédro described a healthy man in this way: “He’s healthy, he’s in shape, when you see him you envy him because he’s healthy” (IDI, MLHIV in treatment, 35–49, San Pédro). Men viewed health in psychological as well as physical terms, for example, as a state of well-being, as expressed by a MLHIV in Abidjan: “Health is peace, health is joy, health is strength” (IDI, MLHIV not in treatment, 25–34, Abidjan).

**Health: Agency.** Men viewed health agency as appearing healthy, which enables participation in social and occupational activities without restriction or stigmatization. HIV threatened men’s health agency if outward manifestations of the disease or the need to take medications for life led to unwanted disclosure.

**Health: Strength.** Strength, as related to health, was expressed in terms of physical ability (e.g., “to walk,” *marcher*) and was contrasted to being bedridden or incapacitated (e.g., “lying down,” *se coucher*). As one MLHIV in treatment said: “As a man, if you have children who work and you yourself are sick and lying at home, if it is the children who must come and provide for you, it is no longer a life in this world. It means that you do not exist anymore, that’s it” (IDI, MLHIV in treatment, 35–49, Bouaké). The physical illness of HIV (including the side effects of ART) threatened men’s health-related strength, preventing them from going about their daily activities.

#### Sexuality

The participants characterized male values of sexuality in several ways, including having multiple sexual partners, having a choice in sexual partners, and procreating. Sexuality overlapped with wealth and social status; wealth allowed for multiple sexual partners; and wealth and women lead to social status. As one participant explained: “Generally, in our environment, a man is said to be successful, based on what? He is healthy, he has a beautiful house, maybe he is polygamous, he has a lot of wives, in any case, that’s it. In our environment, this is what one usually thinks when talking about a successful man” (FGD, man of unknown status, 25–34, Bouaké).

**Sexuality: Agency.** Sexual agency was reflected in the ability to select from multiple potential partners. HIV limits your choices because, as one participant explained: “If you tell her that you have the disease, she will run away” (IDI, unknown status, 35–49, Abidjan). Having a sexually transmitted infection, such as HIV, restricted sexual agency because of a moral obligation to abstain from sex, limit sexual partners, and/or use condoms. Men expressed a responsibility to avoid transmitting disease: “It is better to be the only one contaminated than to contaminate several people at once” (FGD, man of unknown status, 25–34, Abidjan). As such, HIV reduced a man’s agency to seek or have multiple sexual partners. As one participant maintained: “For us, men, HIV is the end of your fun, the end of your joy… it's like you are condemned. When you do not have AIDS, you go to bars, you drink your beer, you find a girl, you go and enjoy… But once you have it, in your neighborhood, if people say you have it, they will point fingers at you” (FGD, man of unknown status, 25–34, San Pédro).

**Sexuality: Strength.** The ability to perform sexually and to procreate reflected sexual strength. HIV threated sexual strength because these men saw it as leading to sexual dysfunction and inhibiting procreation. One man articulated, “A man who is healthy, well, in our jargon when we say a person is a man, we mean he is sexually healthy… When we say a man is ‘in shape,’ well, I’m sorry for the term, but he has a good erection, that’s it” (IDI, MLHIV not in treatment, 35–49, Abidjan). The impact of HIV on procreation was expressed in terms of potential biological infertility—that is, as functional infertility stemming from mandatory condom use and a fear of having children infected with HIV. One participant shared his fears when first diagnosed with HIV, saying, “What worried me at first was to know whether I could still have children” (IDI, MLHIV in treatment, 35–49, Abidjan).

#### Work

Work was described by many participants as “what defines a man.” Along with health, work enabled sexuality, family, and social status. “Work is the first thing, it nourishes the man. In addition, work allows people to see your personality, who you really are,” explained one participant (IDI, man of unknown status, 25–34, Bouaké). A man without work was described as diminished: “The man who does not work is nothing. You do not work, you don’t have money, women will avoid you. You will have no women because you have nothing” (FGD, man of unknown status, 35–49, Abidjan). Men, particularly young men, tended to characterize work and money as foundational to other values, like social status. One participant explained: “Today work has become like a blessing because the end result is money; it gives you a certain social freedom, it gives you a certain social status, it gives you a certain consideration, but in the end, precisely, it is what your work gives you that makes everyone like you and respect you” (FGD, unknown status, 35–49, Abidjan).

**Work: Agency.** Through perceived and real HIV-related stigmatization, HIV threatened agency regarding work. Limited or loss of job opportunities was a significant threat. One participant expressed anxiety about this peril, asking, “How to provide for your family in case the news reaches your workplace and you are fired? Because we know that HIV-positive people are discriminated against in society” (FGD, man of unknown status, 25–34, Bouaké).

**Work: Strength.** HIV presented a direct threat to having the strength to work. According to the interviewees, loss of stamina led to loss of agency: “Consider my situation: you do not have the strength to work… so you have to beg for food. That's the very thing that's going to kill me. Because, in my life, I have pride. I like to share, but I do not like to ask [for help]” (IDI, MLHIV not in treatment, 25–34, Bouaké).

#### Family

Family was another leading value the participants cherished. One participant described the importance of family in the following way:

Family, first of all, starting a family… in society it makes you important. In the eyes of your own family, it makes you important…, since you have your own house and then you have your children that you brought into the world and that you provide for, their food, their health, their schooling, you provide for all their needs. So already, vis-à-vis your peers who have not yet started a family, you are already given respect by society and in your neighborhood. (IDI, man of unknown status, 35–49, Abidjan)

**Family: Agency.** Men described their role as head of the household in terms of decision-making agency. As one participant explained: “I’m the head of the household, but I listen to all of them, from the littlest one to the biggest one, to my wife and to the children. Why? Because I know that I am the head of the household; all the responsibilities are mine, all the decisions are mine” (IDI, MLHIV in treatment, 35–49, Bouaké). HIV threatened a man’s agency with regard to his family, rendering him too sick to make decisions and thus undermining his role as head of the household. The ensuing role-reversal in the family endangered men’s pride and self-concept. As one man said, “I spent six months at first, lying at home. That is not health. Since I was being taken care of by others, that is not living well” (IDI, MLHIV in treatment, 35–49, San Pédro).

**Family: Strength.** The participants discussed the threat of HIV to their position of strength within the family in terms of an inability to look after themselves, their families, and others. Men wanted to avoid dependence and provide for their families, as explained by one MLHIV in treatment:

Well, what is necessary for me is to be self-sufficient, to be able to meet my needs, to be able to have what my heart desires because everyone has desires, everyone would like to become this or that. Anyway, I’m able to be self-sufficient, to provide for my parents, my children and others. For me, this is already a success because I’m able to be self-sufficient, I’m able to take care of myself and my own. (IDI, MLHIV in treatment, 25–34, Abidjan)

A MLHIV not in treatment described the vulnerability provoked by not being in treatment; he explained: “All I care about is being in medical care, being healthy so that I’m able to work. Able to work to make my own living and help others. But if you are not in medical care, not in treatment, you will get sicker and sicker and you will become more vulnerable and it is very serious” (IDI, MLHIV not in treatment, 25–34, Bouaké).

#### Social status

Participants described social status or respect from community as deriving from the attainment of other values—including employment and financial independence, family, procreation and sexual prowess—all of which were underpinned by health. As one participant expressed: “A man is said to be successful when he has a good standard of living. For example, he has a house, he has a car, he is responsible, he is able to feed his family, he is able to help those in need, to take care of his family, it is then that people will say, when he is able to achieve his dreams, then people will say that this person is successful” (FGD, man of unknown status, 25–34, San Pédro).

**Social status: Agency.** Having the agency (through work and financial success) to share resources and assist others further demonstrated and reinforced social status. An MLHIV not in treatment in Bouaké described his vision of a successful man as someone who can help others: “It means having the ability to meet my personal needs and the needs of those around me. And even people I do not know… In passing, when I see someone who is in trouble, I may be able to help him, to demonstrate my love to this person and my spirit of sharing” (IDI, MLHIV not in treatment, 25–34, Bouaké). Participants saw HIV as subverting men’s agency by diminishing their capacity to share resources.

**Social status: Strength.** In the context of this study, strength in social status included having respect and position in the community. As such, a recurring theme was that loss of respect and social rejection due to having HIV would lead to a man’s death more quickly than the disease itself. As a man in a FGD setting said, “It‘s not the disease that kills, it is the rebuff, the rejection, the isolation” (FGD, man of unknown status, 25–34, San Pédro). When describing his fear of HIV testing, one participant explained how he believed the community would regard him and how he would feel if he was diagnosed HIV-positive: “I will not have the same respect as in the past and I won’t be able to bear it” (FGD, man of unknown status, 25–34, Bouaké). Other men portrayed HIV as an illness that shames the man, leading others to judge him: “Because we know that it is a shameful disease, we know how it is caught, so it is obvious why people hide” (FGD, man of unknown status, 25–34, San Pédro).

Finally, HIV threatened men’s self-image and pride. Some MLHIV expressed that HIV was inconsistent with their self-perception. One participant explained: “[I] couldn’t imagine that each time on my way, girls [say], ‘eh he is so cute, he is tall, he is intelligent’ and then, they tell me I have the virus, it is not right, it didn’t sit right with me” (MLHIV not in treatment, 25–34, Abidjan). Participants—especially those who did not feel sick—had difficulty reconciling the way they saw themselves with the way they imagined others saw them given their mental image of an HIV-positive man.

## Discussion

Although most of the men of unknown status in the study were unaware of the treatment eligibility criteria in effect at the time of the study, many participants had outdated information about HIV treatment accessibility and effectiveness and maintained an obsolete interpretation of an HIV-positive diagnosis as a physical and social death sentence. An exploration of the relationships between men’s aspirations and HIV enabled us to explicate how HIV threatens masculine values in ways that exceed the physical or health-specific consequences of HIV. Both HIV-positive men and men of unknown status clearly articulated their trepidation. The threat was internalized as fear around an HIV diagnosis or HIV disclosure. Ultimately, awareness of the inter-relationships of masculinity and HIV can improve the effectiveness of public health messaging and help men overcome perceived barriers to HIV testing.

This study builds on prior work exploring men’s views toward HIV and HIV testing using a gender-sensitive lens [31, 32, 33, 34, 35, 36, 37, 38, 39, 40, 41, 42, 43, 49, 50]. Prior studies describe common themes regarding men’s values and behavior, including the importance of being a provider or demonstrating sexual prowess, toughness, strength, or self-reliance [37, 42, 49]. One notable difference from several prior studies is that the Ivoirian men in this study did not express fear that an HIV-positive diagnosis would reveal infidelity, but rather that their wives or partners might abandon them because of the stigma associated with HIV or the impact of HIV on sexuality, employment, or social status. Further, men expressed delaying testing and treatment not so much because they viewed avoidance as a sign of physical and emotional strength, as Sileo et al. (2018) suggest, but because of fear of the consequences a positive diagnosis would have for other aspirations.

Extant scholarship describes the masculine constructs that emerged from our work–agency and strength–in other ways. A review of seven studies on masculinity and HIV testing in Africa used the terms *strength* and *self-reliance* [42]. In our findings, strength and self-reliance overlapped, so we used strength to describe physical, psychological, and economic strength, power, pride, and invulnerability. The construct of agency in this study also overlaps with self-reliance, but includes independent decision-making—that is, free from external constraints. In addition, the literature described *self-reliance* as “reluctance to seek help from others” which does not completely capture the fear of loss of agency expressed by men in this study.

These insights and a deeper understanding of the relationships between men’s aspirations and HIV may inform the design of more effective social and behavior change programs aimed at increasing male engagement in the HIV care continuum. The Masculine Values Framework posits that (a) personal aspirations are just as, or more, salient to healthy men than specific health concerns; and (b) social and behavior change programs built around a robust, nuanced understanding of men’s aspirations might be more effective than approaches that focus on illness or avoiding illness.

A social and behavior change program based on the Masculine Values Framework might frame HIV testing and ART as a way for MLHIV to continue to aspire to their goals [51]. For example, early detection of HIV and prompt and sustained ART (now available to all people living with HIV for free in Côte d’Ivoire through the universal application of the Test and Treat policy) can help men maintain their physical strength and continue working. A second strategy might be to disseminate messages about the implications of an undetectable viral load and sexual HIV transmission. A third strategy might be to showcase testimonials from MLHIV in treatment living lives fulfilled in the five value domains. These are just a few examples of how the Masculine Values Framework might be applied to improve men’s engagement in the HIV care continuum in Côte d’Ivoire.

This study contributes to the literature on masculinity and HIV by representing the perspectives of men in Côte d’Ivoire as well as their aspirations and fears; however, certain limitations should be taken into consideration. First, we only engaged male participants in the study. The current research does not elucidate the extent to which the five value domains and constructs of agency and strength would resonate among Ivoirian women or the extent to which additional or alternative value domains and constructs would emerge if a similar study were conducted among women. Second, we recruited participants from urban areas only; men in rural areas may have different values. Third, there were many more men in our sample whose HIV status was unknown to investigators than MLHIV. Further, the MLHIV who participated may have differed with respect to their HIV-related attitudes and experiences from those who did not. Finally, the applicability of the Masculine Values Framework may be limited to the Ivoirian context.

## Conclusions

This study contributes to the scholarship on the intersections of masculinity and HIV. Through the voices of Ivoirian men, we constructed a framework that depicts how HIV threatens men’s agency and strength with respect to their health, sexuality, work, family, and social status. The Masculine Values Framework provides insight into the widespread resistance among men in Côte d’Ivoire to HIV testing and brings men’s fear into sharper focus. Through a more nuanced understanding of why men avoid HIV testing—beyond the health implications—social and behavior change programs can design better interventions to help men overcome barriers to engaging in the HIV care continuum and help Côte d’Ivoire reach the 90-90-90 targets.

## Supporting information

S1 COREQ Checklist (PDF)

S1 Fig (TIFF)

S1 IDI and FGD Guide English. (DOCX)

S2 IDI and FGD Guide French. (DOCX)

S1 Related Manuscript (PDF)

S2 Related Manuscript (PDF)
