# Supplementary figures and images for: Bringing fear into focus: The intersections of HIV and masculine gender norms in Côte d’Ivoire
Source: PLoS One. 2019 Oct 23;14(10):e0223414. doi: 10.1371/journal.pone.0223414 (PMC6808548; doi:10.1371/journal.pone.0223414)

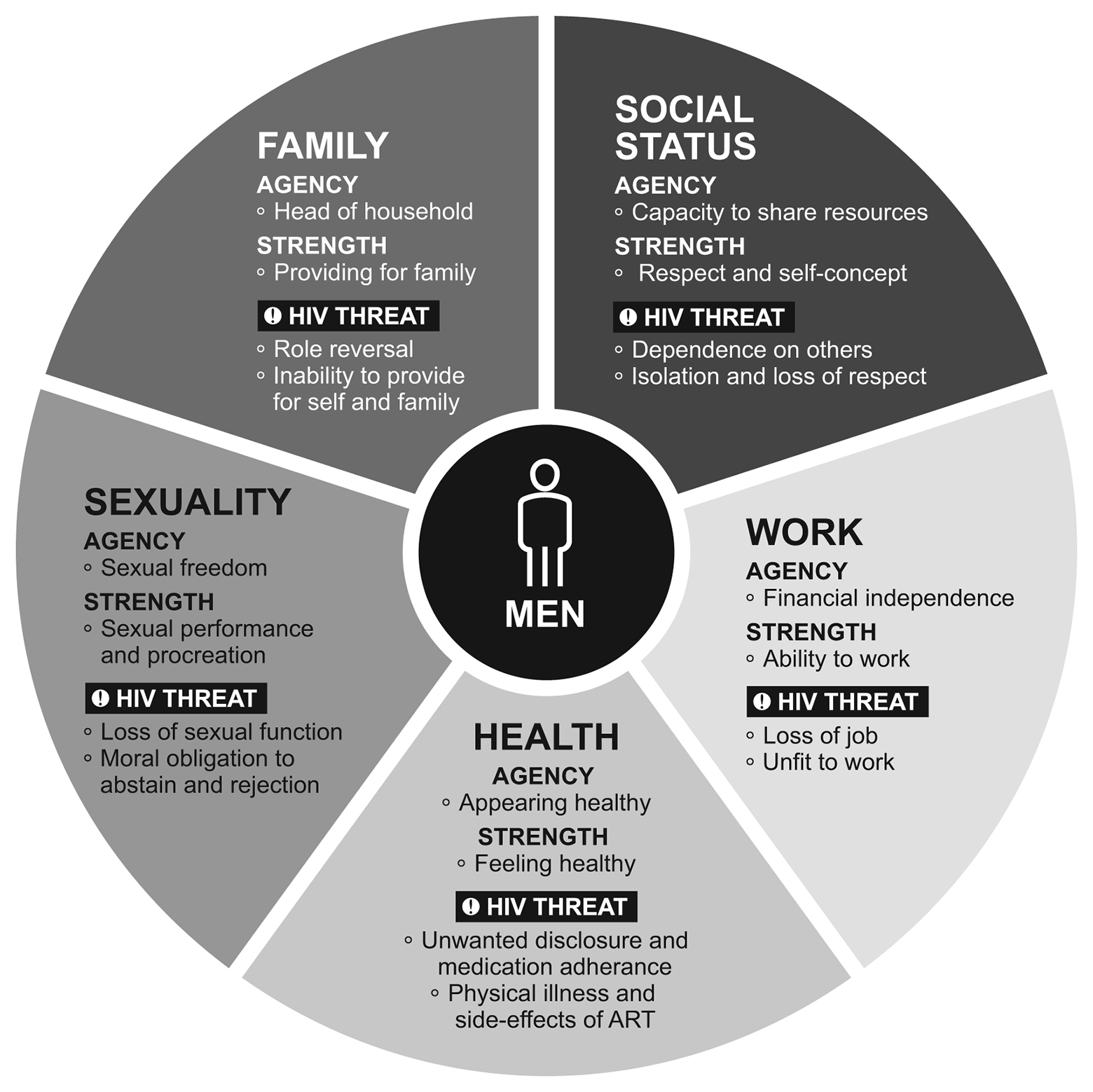

Supplement: S1 Fig — (TIFF) [file pone.0223414.s002.tiff]
