# Supplementary material for: Bringing fear into focus: The intersections of HIV and masculine gender norms in Côte d’Ivoire
Source: PLoS One. 2019 Oct 23;14(10):e0223414. doi: 10.1371/journal.pone.0223414 (PMC6808548; doi:10.1371/journal.pone.0223414)
Supplement: S1 IDI and FGD Guide — (DOCX) [file pone.0223414.s003.docx]

**In-depth interview: Adult men**

**Introduction**

**(Time approximately 1 hour)**

After conducting the consent process and getting permission to audio-record the interview, start the interview with an introduction.

***Thank you for talking with me today. The information you provide will remain completely confidential and will be helpful to improve health services (especially around HIV and sexual health) for men.***

The following provide general instructions on conducting the interview.

The questions below are starting points for key topics. These are not a set of questions to ask nor does it contain the specific language best for each group of individuals. Extensive discussion about one topic is more valuable then superficial discussion of all the themes included here.

To ease the respondent into the interview it would be important to build some rapport with them by asking simple questions such as ‘How are you today?’

Many of these questions are focused around perceptions of health, health care, and HIV. Questions can be directed either at the participant’s view or what he thinks other similar men would think. Sometimes describing what others may think allows a participant to be more open about their own views.

| **INTERVIEW GUIDE FOR PARTICIPANTS WHOSE HIV STATUS IS UNKNOWN TO THE INTERVIEWER** | | | |
| --- | --- | --- | --- |
| **Theme** | **Questions** | | |
| **Demographics** | Without telling me your name, please introduce yourself by telling me your age, education level, marital status, and profession. | | |
| **Aspirations** | What do you value most in life? Try to think of the 3-5 most important.  For each value,  What does [value] mean to you?  What do you do now to achieve [value]?  What do you think about others who have [value]?  What will you do in the future to achieve [value]?  What would make you feel successful in life? | | |
| **Health** | ***[If health was not among the 5 most important values].*** How does health relate to the values you listed? What does health mean to you? What can a healthy person do? What sorts of things indicate that someone does not have health? What do you do now to achieve health? What will you do in the future to achieve health? What are your biggest health concerns or needs? | | |
| **Health and Gender** | Question 1 | Question 2 | Question 3 |
|  | Describe a man who is in good health. What does a healthy man look like?  Describe a woman who is in good health. What does a healthy woman look like?  What are the differences in what health means for you compared to how you think of health for a women? What are the similarities? | What do you do to protect your health and well-being?  What do women you know do to protect their health and well-being? | Do you ever take vitamins or strength boosters?  If so, why?  Do you ever take medications? If so why?  Do you take any medications regularly over months? What is the reason for taking these medications? |
| **HIV** | Question 1 | Question 2 | Question 3 |
|  | What does HIV mean to you? Explain more about what you mean. How does someone find out that he has HIV? | If you learn that a man you know has HIV, what do you think? Which men get HIV? | What should a man do if he thinks he has a sexually transmitted disease?  What should a man do if he has other sexual concerns? (sexual performance) |
| **HIV Testing** | What is an HIV test?  *[If the participant does not understand the word “depistage”, the French word for HIV test, explain that it is a blood test that detects whether a person has HIV or not]*  Who should get tested for HIV?  Why might a man want to get tested for HIV? Why might a man not want to get tested for HIV? Why might a woman want to get tested for HIV? Why might a woman not want to get tested for HIV?  After the HIV test, what does it mean if you’re told that you have HIV? How would you react? What does it mean if you’re told that you do not have HIV? How would you react? | | |
| **Test & Start** | Scenario | | |
|  | **Paul is a man with a wife and 2 children who works at an industry. He went for an HIV test today and was told that he has HIV.** What do you think is going through his mind? What do you think he might do next? Who do you think he might speak with?  **The testing counsellor asks him to come back to see the doctor.** What would you think if you were Paul? Should Paul come back to see the doctor? Why or why not?  **Paul goes to the doctor and is given HIV medications to be taken every day before bed time.** Should he take them? Why or why not? | | |
| **HIV treatment** | What have you heard about HIV treatment? (reasons to take, problems) | | |

**In-depth Interview: Adult Men, HIV-positive**

**Introduction**

**(Time approximately 1 hour)**

After conducting the consent process and getting permission to audio-record the interview, start the interview with an introduction.

***Thank you for talking with me today. The information you provide will remain completely confidential and will be helpful to improve health services (especially around HIV and sexual health) for men.***

The following provide general instructions on conducting the interview.

The questions below are starting points for key topics. These are not a set of questions to ask nor does it contain the specific language best for each group of individuals. Extensive discussion about one topic is more valuable then superficial discussion of all the themes included here.

To ease the respondent into the interview it would be important to build some rapport with them by asking simple questions such as ‘How are you today?’

Many of these questions are focused around perceptions of health, health care, and HIV. Questions can be directed either at the participant’s view or what he thinks other similar men would think. Sometimes describing what others may think allows a participant to be more open about their own views.

| **INTERVIEW GUIDE FOR HIV POSITIVE PARTICIPANTS** | | | | |
| --- | --- | --- | --- | --- |
| **Theme** | **Questions** | | | |
| **Demographics** | Without telling me your name, please introduce yourself by telling me your age, education level, marital status, and profession. | | | |
| **Aspirations** | What do you value most in life? Try to think of the 3-5 most important.  For each value,  What does [value] mean to you?  What do you do now to achieve [value]?  What do you think about others who have [value]?  What will you do in the future to achieve [value]?  What would make you feel successful in life? | | | |
| **Health** | ***[If health was not listed among the most important values].*** How does health relate to the values you listed? What does health mean to you? What can a healthy person do? What sorts of things indicate that someone does not have health? What do you do now to achieve health? What will you do in the future to achieve health? What are your biggest health concerns or needs? | | | |
| **Health and Gender** | Question 1 | Question 2 | | Question 3 |
|  | Describe a man who is in good health. What does a healthy man look like?  Describe a woman who is in good health. What does a health woman look like?  What are the differences in what health means for men compared to women? What are the similarities? | What do men like you do to protect their health and well-being?  What do women in your community do to protect their health and well-being? | | What are some physical problems you are concerned about or have had?  From whom do you seek advice, guidance? (male friends, health advises / healers, doctors, nurses) |
|  |  |  |  |  |
| **HIV** | What does HIV mean to you? Explain more about what you mean.  Tell us about whether you have told anyone about having HIV? If YES, who did you tell? Describe the experience. If NO, what has prevented you from sharing your status? | | | |
| **HIV Testing** | How did you learn you had HIV?  Why did you get tested?  Describe what happened after you received your HIV test results. | | | |
| **HIV Positive** | What treatment do you receive currently? What is the goal of your treatment? How do you know whether or not the treatment is working? | | | |
| **Knowledge about HIV treatment** | What treatments are available for HIV here? For whom are they available?  What is the purpose of the treatment?  Have you ever heard of CD4 count? If so, explain what it means to you?  Have you ever heard of viral load? If so, explain what it means to you?  What about an undetectable viral load? What does that mean? | | | |
| **Test & Start** | Question 1 | | Question 2 | |
|  | Some people with HIV get treatment and some do not. What do you think about this? What does treatment for HIV mean to you? | | What would you think if everyone who tests positive for HIV received systematic treatment? What would other people think if everyone with HIV received systematic treatment? | |
| ***HIV Positive** | ***If on ART:*** What motivated you to start treatment?  ***If not on ART:*** Do you want to start treatment? Why / why not?  What motivates you or others like you to stay in treatment?  What makes it difficult for you or others like you to stay in treatment?  What might cause you or others like you to abandon treatment?  What additional sorts of things do you want to know about treatment? | | | |

**Focus Group Discussion: Adult Men, HIV status unknown to facilitator**

**Introduction**

**(Time approximately 1.5 hours)**

The questions below are starting points for key topics. These are not a set of questions to ask. Extensive discussion about one topic is more valuable then superficial discussion of all the themes included here.

To ease the respondents into the group discussion it would be important to build some rapport with them by asking simple questions such as ‘How are you today?’

Many of these questions are focused around perceptions of health, health care, and HIV. Questions can be directed either at the participant’s view or what he thinks other similar men would think. Sometimes describing what others may think allows a participant to be more open about their own views.

The goal is to identify and describe community norms and themes around these topics and not to understand nuance from any single group member.

**Starting the interview**

Start with appreciation of the participant:

***“Thank you for talking with me today. The information you provide will remain completely confidential and will be helpful to improve health services (especially around HIV and sexual health) for men.”***

Each topic/question should lead to a group discussion. The group views are important, not each individual’s views.

| **FOCUS GROUP DISCUSSION GUIDE FOR PARTICIPANTS WHOSE HIV STATUS IS UNKNOWN TO THE INTERVIEWER** | | | |
| --- | --- | --- | --- |
| **Theme** | **Questions** | | |
| **Demographics** | Without telling me your name, please introduce yourself by telling me your age, education level, marital status, and profession. | | |
| **Aspirations** | What do men in this community value most in their life? Try to think of the 3-5 most important.  For each value,  What does [value] mean in the community?  How is [value] achieved?  What makes a successful man? | | |
| **Health and Gender: Men** | ***[If health was not listed among the most important values].***  How does health relate to the values for men in this community?  What does health mean to you and how do you preserve your health?  What are some common ailments that men get?  What do men do to prevent from getting such ailments?  What do men do to treat such ailments? | | |
| **Health and Gender: Women** | And for women, what does health mean for them?  What are some common ailments that women get?  What do women do to prevent from getting such ailments?  What do women do to treat such ailments? | | |
| **HIV** | Question 1 | Question 2 | Question 3 |
|  | What does HIV mean to men in this community? Explain more about what you mean.  Which men are at the greatest risk for HIV? | How do you think a man finds out that he has HIV? | If a man has HIV, should he tell his wife? Why or why not? Should he tell his girlfriend? Why or why not? |
| **HIV Testing Advantages & Disadvantages** | What is an HIV test?  *[If the participants do not understand the word “depistage”, the French word for HIV test, explain that it is a blood test that detects whether a person has HIV or not]*  Who should get tested for HIV?  Why might a man want to get tested for HIV? Why might a man not want to get tested for HIV?  After the HIV test, what does it mean if you’re told that you have HIV? How would you react? What does it mean if you’re told that you do not have HIV? How would you react? | | |
| **Test & Start** | Scenario | | |
|  | **Paul is a man with a wife and 2 children who works at an industry. He went for an HIV test today and was told that he has HIV.** What do you think is going through his mind? What do you think he might do next? Who do you think he might speak with?  **The testing counsellor asks him to come back to see the doctor.** What would you think if you were Paul? Should Paul come back to see the doctor? Why or why not? What does the group think?  **Paul goes to the doctor and is given HIV medications to be taken every day before bed time.** Should he take them? Why or why not?  Do you know anyone with HIV?  Do you know if they are getting treatment? If not: What do you think about that? If yes: How do you think treatment is helping him or her? | | |
| **Knowledge about HIV treatment** | What treatments are available for HIV here? For whom are they available?  What is the purpose of the treatment?  Do you think treatment should be offered to everyone with HIV? Why or why not?  What have you heard about blood tests people living with HIV get? What is a CD4 count? What does viral load mean to you? What does an undetectable viral load mean to you? | | |
