# Supplementary material for: Bringing fear into focus: The intersections of HIV and masculine gender norms in Côte d’Ivoire
Source: PLoS One. 2019 Oct 23;14(10):e0223414. doi: 10.1371/journal.pone.0223414 (PMC6808548; doi:10.1371/journal.pone.0223414)
Supplement: S1 Related Manuscript — (PDF) [file pone.0223414.s005.pdf]

AIDS and Behavior
https://doi.org/10.1007/s10461-019-02614-5

ORIGINAL PAPER

"You Take Medications, You Live Normally": The Role of Antiretroviral Therapy in Mitigating Men's Perceived Threats of HIV in Côte d'Ivoire

Zoé Mistrale Hendrickson[1,2,6] · **Danielle A. Naugle**[1] · **Natalie Tibbels**[1] · **Abdul Dosso**[3] · **Lynn M. Van Lith**[1] · **Elizabeth C. Mallalieu**[1] · **Diarra Kamara**[3] · **Patricia Dailly-Ajavon**[3] · **Adama Cisse**[3] · **Kim Seifert Ahanda**[4] · **Sereen Thaddeus**[4] · **Stella Babalola**[1,2] · **Christopher J. Hoffmann**[5]

© Springer Science+Business Media, LLC, part of Springer Nature 2019

Abstract
Men diagnosed with HIV face gender-related barriers to initiating and adhering to antiretroviral therapy (ART). This qualitative study (73 in-depth interviews; 28 focus group discussions), conducted with men in three urban sites in Côte d'Ivoire in 2016, examined perceptions of ART, including benefits and challenges, to explore how ART mitigates HIV's threats to men's sexuality, economic success, family roles, social status, and health. Participants perceived that adhering to ART would reduce risk of transmitting HIV to others, minimize job loss and lost productivity, and help maintain men's roles as decision makers and providers. ART adherence was thought to help reduce the threat of HIV-related stigma, despite concerns about unintentional disclosure. While ART was perceived to improve health directly, it restricted men's schedules. Side effects were also a major challenge. Social and behavior change approaches building on these insights may improve male engagement across the HIV care continuum.

Keywords Masculinity · HIV · Antiretroviral therapy · Côte d'Ivoire · West Africa

Resumen
Hombres seropositivos enfrentan barreras de género para iniciar y adherirse al tratamiento antirretroviral (TAR). Este estudio (73 entrevistas en profundidad; 28 grupos focales), realizado en 2016 en Costa de Marfil con hombres en tres centros urbanos, examinó percepciones sobre TAR, beneficios y retos, para explorar cómo éste mitiga amenazas a la sexualidad, éxito económico, roles familiares, estatus social y salud del hombre. Participantes entendieron que TAR reduciría riesgo de transmitir VIH, minimizaría pérdida de empleos, productividad, y ayudaría a preservar su rol como tomadores de decisiones y proveedor. Adherencia al TAR se entendió como medio para reducir estigma del VIH, no obstante preocupaciones sobre divulgación involuntaria. TAR se entendió como forma de mejorar la salud pero restringiendo actividades del hombre. Efectos

✉ Zoé Mistrale Hendrickson
zhendri1@jhu.edu

1 Johns Hopkins Center for Communication Programs, Baltimore, MD, USA

2 Health Behavior & Society Department, Johns Hopkins Bloomberg School of Public Health, Baltimore, MD, USA

3 Johns Hopkins Center for Communication Programs, Abidjan, Côte d'Ivoire

4 United States Agency for International Development, Washington, DC, USA

5 School of Medicine, Johns Hopkins University, Baltimore, MD, USA

6 111 Market Place, Suite 310, Baltimore, MD 21202, USA

Published online: 31 July 2019

secundarios resaltaron como gran reto. Utilizando estos hallazgos, enfoques de cambio social y de comportamiento pueden mejorar participación del hombre en el continuo de atención del VIH.

Introduction

Prompt initiation of antiretroviral therapy (ART) following an HIV diagnosis has the potential to improve individuals' long-term health outcomes and reduce HIV incidence globally [1]. Ensuring medication adherence and retention in care are also essential to addressing the HIV epidemic [2, 3]. To increase HIV-positive individuals' knowledge of their HIV status, initiation of ART, and achieve viral suppression—thereby meeting UNAIDS' 90-90-90 targets [1]—countries around the world are implementing Test and Treat (universal treatment) programs. Such programs are designed to increase individuals' access to and utilization of HIV services [4, 5] and increase engagement along the HIV care continuum. In Côte d'Ivoire, a Test and Treat policy was adopted in February 2017 to increase prompt initiation of ART.

With an HIV prevalence of 2.7% [6], Côte d'Ivoire has one of the highest burdens of HIV in West Africa. A robust national program has sought to increase HIV diagnosis and ART initiation [7]. However, uptake of testing and treatment services among specific populations has lagged. In Côte d'Ivoire as well as elsewhere in sub-Saharan Africa, men have lower testing and ART initiation rates than women [2, 8–15] and often have reduced survival once on ART as a result of delays in treatment initiation and poor retention in care [14, 16, 17].

Barriers to men's engagement in the HIV care continuum exist at multiple socio-ecological levels. For men, individual attitudes and perceptions, interpersonal relationships, health facility characteristics, community norms, and additional social and structural factors influence uptake of HIV testing and treatment services [18–20]. Specific barriers include fears of HIV and status disclosure, concerns about side effects, limited access to facilities (e.g. working during hours of operation, distance, or prioritization of care for women over men), HIV-related stigma, and the impact of a positive HIV diagnosis on a man's self-confidence, social status, or expected role as family provider [2, 10, 12, 13, 17, 21–24].

Studies in sub-Saharan Africa, and elsewhere, have emphasized how these differences between women and men are linked to domains of masculinity—such as agency, strength, or respectability—and gender roles that affect men's engagement in risk behaviors and decisions about whether to get tested, disclose their status, or seek care. Treating HIV may also be considered a threat to a man's strength or independence, contributing to delays in initiating treatment and non-adherence to ART [8–10, 25–32]. Building on other findings from sub-Saharan Africa, Naugle et al.'s [33] formative research in Côte d'Ivoire identified the important role of five value domains in men's self-conceptions—sexuality, family, work, social status, and health—and illustrated how HIV threatens men's agency and strength across each of these value domains.

In 2016, program data showing major challenges to HIV testing, treatment initiation, and viral suppression among men in Côte d'Ivoire led the U.S. President's Emergency Plan for AIDS Relief (PEPFAR) team in Côte d'Ivoire to identify a need for formative research to improve future PEPFAR programmatic approaches for reaching men. According to the 2011–2012 Demographic and Health Survey, 7.8% of new HIV infections occurred among men over the age of 35, and this number was found to be increasing. Sixty percent of men were unaware of their HIV status [34]. Stigma, late presentation for treatment, high loss to follow-up, difficulty with disclosure, denial of HIV status, and risky sexual practices were among those factors that led PEPFAR to seek a more in-depth understanding of the barriers to HIV testing and treatment for men. In response to this need, in-depth interviews (IDIs) and focus group discussions (FGDs) were conducted in 2016 to understand men's perspectives on HIV testing and treatment, specifically the barriers men experience in getting tested and subsequently seeking treatment for HIV. Results were also used to inform development of the Johns Hopkins Center for Communication Program's Brothers for Life Plus (BFL+) program, which is designed to improve the health and wellbeing of men and their families with a focus on the multi-level factors influencing HIV prevention, testing, and treatment initiation in Côte d'Ivoire [35].

Here we draw on Naugle et al.'s [33] Masculine Values Framework (MVF; Fig. 1) and the formative research described above to understand Ivoirian men's views of ART, within the context of the MVF, in order to develop more effective social and behavior change (SBC) strategies. Building on the gender-related barriers to getting tested as well as initiating and adhering to treatment, this article explores Ivoirian men's perceptions of ART and how ART intersects with men's values. Specifically, we sought to identify (1) gaps in understanding of treatment and (2) the potential for ART to mitigate HIV's threats to these core value domains.

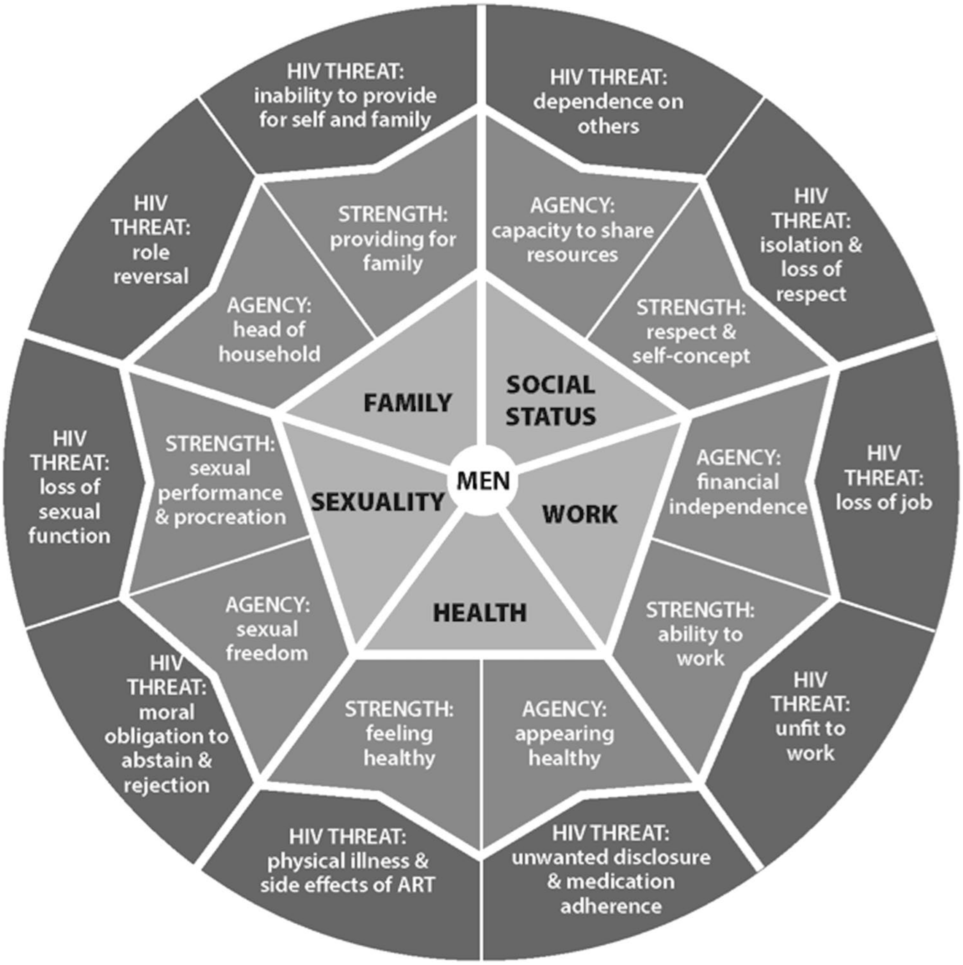

Fig. 1 Naugle et al.'s [33] Masculine Values Framework (MVF) of men's core values, relevant masculinity constructs, and the potential threats of HIV across key value domains. Reproduced with permission from Naugle et al. [33]

Methods

Setting and Participants

Seventy-three IDIs and 28 FGDs were conducted in three urban sites across Côte d'Ivoire—Abidjan at Yopougon-Ouest-Songon, Bouaké, and San Pédro—where HIV prevalence remains high [34]. While all sites were urban, they varied in size as well as population. Abidjan, located on the coast, is the largest city in Côte d'Ivoire, with a population of nearly five million [36], and is the economic capital of the country. San Pédro's population rose substantially in the second half of the twentieth century after the construction of a port that continues to attract internal migrants. Economic activities in San Pédro attract workers from across Côte d'Ivoire as well as elsewhere in West Africa. Located between Abidjan and Ouagadougou, Burkina Faso, Bouaké is a city in the central area of Côte d'Ivoire where agricultural production (e.g. cotton and tobacco), textiles, and trade are important aspects of the economy. As a result, it is an important transit point for West African and international traders [37–42]. While Test and Treat was adopted in February 2017, it was not in place during data collection for this study. Prior to Test and Treat, HIV treatment eligibility in Côte d'Ivoire was based on a CD4 count of 500 cells/mm^3 or lower, presence of active Tuberculosis, or pregnancy. ART was provided free of charge to those who were eligible. People with CD4 counts greater than 500 cells/mm^3 were monitored to treat opportunistic infections and provide prophylactic treatments as appropriate.

Participants were men between 25 and 49 years of age recruited from one of three groups: those whose HIV status was unknown to investigators, men living with HIV (MLHIV) in treatment, or MLHIV not in treatment. IDIs were conducted with MLHIV to understand their lived experiences testing for and being diagnosed with HIV. IDIs with men whose HIV status was unknown to investigators explored their perspectives on HIV testing and treatment. In addition, FGDs were conducted only with men whose HIV status was unknown to investigators to explore community and social norms around HIV, HIV testing, and treatment seeking. FGDs were not conducted among MLHIV to maintain confidentiality. Due to the study's larger objective of understanding barriers men face in getting tested for

HIV, men whose HIV status was unknown to investigators comprised the largest proportion of study participants. We drew on both the perceptions of men whose HIV status was unknown to the researchers and the experiences of men living with HIV in this article to analyze the potential effects of ART on men's perceived threats of HIV. This research study was approved by the Johns Hopkins Bloomberg School of Public Health's institutional review board (IRB#00007374) as well as the local ethical review committee (Comité National d'Ethique de la Recherche) in Côte d'Ivoire.

Recruitment and Data Collection

Active participant recruitment took place through non-governmental, community-based organizations, men's social groups (*grins*), and men's workplaces, which included agricultural as well as industrial sectors. MLHIV on treatment and not on treatment were recruited through non-governmental organizations (NGOs) working specifically with people living with HIV, while participants whose HIV status was unknown to interviewers were recruited through community leaders and men's social groups. In-person recruitment was facilitated using a recruitment script that outlined the objectives of the study for prospective participants. Trained qualitative interviewers conducted the IDIs and FGDs in private rooms in schools, office buildings, or health facilities. Male Ivoirian interviewers conducted all interviews and FGDs to help make male participants comfortable discussing sexual and relationship topics. The interview guides covered topics such as men's values and aspirations; perceptions and experiences of general care-seeking; knowledge, beliefs, perceived social norms, and experiences with or understanding of HIV testing and treatment; and, for MLHIV, experiences engaging in the HIV continuum of care. The recruitment and consent process as well as all interviews and FGDs were conducted in French. All interviews and FGDs were audio-recorded, and transcribed verbatim in French. Informed consent was obtained from all individual participants included in the study. Three authors (ZH, NT, and DN) listened to twenty percent of the audio-recordings of interviews and compared them with the written transcripts to ensure that the transcripts were verbatim and complete.

Analysis

The research team coded and analyzed the transcripts in French using ATLAS.ti software [43]. A preliminary codebook was developed based on the literature on multilevel barriers and facilitators to engaging in the HIV continuum of care (deductive codes) and was later adapted to reflect emergent themes from the transcripts (inductive codes). Five members of the research team participated in the coding process, with 20 percent double-coded for consistency. Coders also drafted memos during the coding process to capture complete participant stories and "real time" researcher insights.

As outlined by Naugle et al. [33], a thematic analysis explored coded transcripts to develop the Masculine Values Framework (MVF). As shown in Fig. 1, Naugle et al. [33] demonstrated how men's HIV testing and treatment seeking were affected by the extent to which HIV threatened men's sexuality, work and financial success, family roles, social status, and health. Each of these value domains was expressed in terms of agency (how men described independence and the capacity to control their own lives) and strength (both physical and psychological; Fig. 1).

Evident throughout Naugle et al.'s [33] analysis was the potential for ART to mitigate many of the threats identified by men. Building on these findings and drawing on the barriers identified by Naugle et al. [33] in the MVF, we then conducted a subsequent deductive thematic analysis focused on perceptions of ART that was organized by key domains of the MVF. We examined the nuances of men's understandings and perceptions of ART. In this article, we build on Naugle et al.'s [33] findings to explore the extent to which men's perceptions of ART intersect with Ivoirian men's core values and the potential for ART to mediate the threats of HIV to these values.

Results

Across the three sites, 15 IDIs were conducted with MLHIV in treatment and 13 with MLHIV not yet in treatment. As men were interviewed for this study before the rollout and expansion of Test and Treat in Cote d'Ivoire, most MLHIV not yet in treatment were ineligible for treatment at the time of the interview. A few had started treatment but had stopped. Forty-five IDIs and 28 FGDs were conducted among men whose HIV status was unknown to the research team. Most participants were in the 35–49 age group (Table 1).

In the sections below, we describe participants' knowledge and understanding of HIV treatment and men's perceptions of ART, including its perceived benefits and challenges, by their relevance to the five masculine value domains described in the MVF (Fig. 1).

Men's Understanding of ART

Men frequently described how ART's ability to ameliorate physical symptoms was one of the major benefits of treatment. Often, the goal of treatment was described in terms such as: "to improve one's health condition;" "to live longer;" "to avoid death;" "to weaken the microbes;" "to delay the evolution of the disease;" "in order not to be sick;"

Table 1 Details of men participating in IDIs and FGDs as part of this qualitative study

Participant type	Number of IDIs		Number of FGDs (note: 4–8 participants per FGD)		Total number of participants
	25–34 years old	34–49 years old	25–34 years old	34–49 years old	
Men whose HIV status was unknown to investigators	19	26	12	16	199 (45 IDIs, 154 in FGDs)
Men who were HIV-positive and in treatment	6	9	–	–	15
Men who were HIV-positive but not in treatment	7	6	–	–	13
Total number of participants					227

or “to be in good shape.” Success of treatment was often described in terms of symptoms rather than abstract laboratory definitions based on CD4 count or viral load measures. In fact, the connections between the effects of ART and viral suppression or CD4 count were often not well understood. While some men described the goals of treatment as “to make the virus dormant” or “to reduce the level of the virus in the blood,” responses like these were less common. One man 35–49 from a FGD in San Pédro said, “it is when there is a lack of something in the blood that… I have forgotten a little it’s been a long time since I have done [learned] that” (FGD, man of unknown status, 35–49, San Pédro).

While not reported by the majority of participants, several men had the false assumption that ART was meant to be a cure for HIV. For example, in the following quotation one man emphasized how—in his opinion—ART cures HIV. He said,

> …I learned that treatment now has a curative purpose. I have not confirmed it yet, but I learned that all those who are placed on treatment, um retrovirals, um antiretrovirals, those who follow the treatment as expected over a certain number of years, they are cured of the disease because I … since I am not a doctor I can’t get into the details, but I know that I learned that those, that all those that put themselves, that follow their treatment correctly, they can be cured after several years (FGD, man of unknown status, 35–49, Bouaké).

The potential impact of this perception was illustrated in a testimonial from a MLHIV not on treatment. “That’s when I stopped the treatment. I was in good shape, very fat, and the sweat would trickle down my body. I worked hard, played football, so I thought I was already cured” (IDI, MLHIV not in treatment, 35–49, San Pédro).

The value of accurate and up-to-date treatment information, in conjunction with adherence to medication, was, however, summarized by a man with higher treatment literacy participating in an IDI, who said,

> Yes, I’ve heard of CD4 because every time when they do the check-up, they tell us that our CD4 count is down or it is up, they always tell us to make an effort so that it can go up because when it goes up, in any case you’re all set but when it goes down, it… opens the doors and then opportunistic diseases come to bother us, that’s what often makes us sick. But when you take care, you eat well, you take your medications, I think that opportunistic diseases don’t take root. … [If the CD4 count goes up] that means that the disease, the virus even, it starts to weaken but it’s when it [CD4 count] goes down that the virus takes over so you have to do everything possible so that the virus does not increase (IDI, MLHIV in treatment, 35–49, Abidjan).

Knowledge and understanding of treatment interacted with men’s engagement at all stages of the HIV care continuum. Participants described how if someone were unaware that HIV could be treated, they might be reluctant to test in the first place as they continued to perceive HIV to be a death sentence. Furthermore, some men who considered ART as a cure for HIV stopped treatment or failed to acknowledge the importance of the medication schedule as their symptoms subsided. Ultimately, men’s perceptions of HIV and its treatment relied on their understanding of the goals of HIV treatment and knowledge of ART, and these understandings varied. For example, participants who provided more detailed descriptions of CD4 count and viral load were often MLHIV, but not all MLHIV described CD4 cells in the same way or knew what they were. While some MLHIV had greater knowledge of HIV and HIV treatment than other participants, there remained a wide range of HIV and ART knowledge among MLHIV and men whose status was unknown to investigators.

Men's Perceptions of the Benefits, or Challenges, of ART

Men described the real and potential benefits of ART to their sexuality, work and financial success, family, social status, and health. Within each domain below, we outline participants' perceptions of ART and map these perceptions onto the MVF to illustrate the potential that each perceived benefit, or challenge, has to address the threats of HIV to men's core values.

Sexuality

Some men perceived that ART could reduce the risk of transmitting HIV to their sexual partner(s), thereby making it sexually liberating and restoring men's sexual agency. In a FGD among men with unknown HIV status, one man explained how ART enabled men to have sex with their partners with reduced risk of transmission. He said,

> So, when you are given the drug, as time passes, the amount of virus decreases, it decreases until it may become undetectable. And people might say that this gentleman here, he's moderately healthy, you can have sex with your wife without a condom in order to have children (FGD, man of unknown status, 25–34, San Pédro).

At the same time, men also reported that ART reduced the risk of transmitting HIV to a future child. As one MLHIV who was not yet in treatment explained, "What makes me want to be in treatment, is my health…to not contaminate others and to protect the children that I should have" (IDI, MLHIV not in treatment, 25–34, San Pédro). While only evoked by a few participants in interviews and FGDs, ART was seen—by those that were aware of treatment as prevention of onward transmission—to be restorative of this key aspect of sexuality.

Work and Financial Success

ART allowed men to uphold a healthy appearance and live and work normally. As a man whose HIV status was unknown said, "Even so, you have HIV, you can work, you can go to work as you like. You take medications, you live normally" (IDI, man of unknown status, 35–49, Abidjan). Another participant, a MLHIV in treatment, said, "When I take the treatment, it gives me strength again… to go about my business." (IDI, MLHIV, in treatment, 25–34, San Pédro). By maintaining or restoring health, ART was thought to provide men with the physical strength for work. While participants diagnosed with HIV and those with unknown status discussed the value of work, MLHIV in particular discussed concerns about the effects of HIV on their ability to work. As described in the *Health* section below, concerns about the burden of medication regimens and how side effects associated with ART might impact one's work were also raised by participants.

Family

Men perceived that ART enabled them to fulfill their role as the head of the household by taking care of their family members and their materials needs. ART helped men to remain healthy enough not to become dependent on their families. This was viewed as helping them to maintain their role as the head of the household with responsibilities as the family's provider and decision-maker. As one MLHIV currently in treatment said:

> When you see someone you can tell they have AIDS, he even has difficulty eating, you know that he, he is not healthy… Yeah today, I am healthy, I tell myself I am healthy today in comparison to the past, but today I can eat well, I can take care of my business so that I can take care of my little family (IDI, MLHIV in treatment, 35–49, San Pédro).

ART was therefore intimately linked with men's roles and responsibilities in their families.

Social Status

The ability to treat HIV using ART was considered an important way through which social stigma related to HIV could be mitigated. Before the roll-out of Test and Treat in Côte d'Ivoire, one MLHIV who was not in treatment explained how if everyone had access to treatment, HIV-related stigma might be reduced. He said,

> If everybody has the treatment, if the majority follow the treatment, and they are fine, if someone else has it [HIV], he's not going to hide any more. He will say ah you, you have it, you hide it, it can be treated, it's ok, I myself have it. You'll have the courage to tell people. That's it. (IDI, MLHIV not in treatment, 25–34, Abidjan).

At the same time, men perceived that ART could help them maintain their social status by avoiding unwanted disclosure related to visible manifestations of HIV including opportunistic infections and weight loss. As one MLHIV in treatment said, "To not just get sick and then realize tomorrow that, 'ah, I have HIV,' and then 'now people will be discouraged about me.' That is what pushes me to come take the medication" (IDI, MLHIV in treatment, 25–34, Abidjan). While not actively addressing social stigma associated with HIV, men considered ART to be a way to prevent unintentional disclosure of one's status and maintain their current social status.

Despite these potential benefits of ART, stigma and unintentional disclosure also remained major concerns for men regarding ART. Men described fears of being seen accessing care at a health facility or of others seeing their medications. In light of these contradictions, men's perceptions of ART, including its benefits and challenges, demonstrated how ART initiation and adherence could intersect both positively and negatively with one's social status.

Health

Participants considered ART to have both physical and psychological effects. As one man mentioned in a FGD in Bouaké, "it treats both aspects, it will considerably slow down the progression of this disease, but it also cures you psychologically" (FGD, man of unknown status, 25–34, Bouaké). The appearance of good health was a major benefit of ART. As one MLHIV in treatment recounted, "He was skinny, indeed, he was not nice to look at. But since he started treatment, he has become 'zooo' as we say in our language, attractive" (IDI, MLHIV in treatment, 25–34, Bouaké). This promise of feeling and looking healthy motivated some men to start and adhere to treatment. As one participant said, "With the treatment, I feel normal as if I was not sick or anything." (IDI, MLHIV in treatment, 25–34, Abidjan). The return of normality with ART was a shared perception.

Nonetheless, participants expressed some health-related concerns about ART. Several participants understood that ART required long-term, lifelong commitment. They emphasized the lifelong changes in habits that were necessary to adhere to ART. The daily medication schedule was described negatively and often seen as restrictive, thereby threatening men's independence. In the following quotation, a man from Bouaké explained men's resistance to taking medications routinely over time. He said:

> Yes, the treatment, see, really the information that I have received, like I said, really it is difficult to learn. You know men don't really like to change their habits. Someone who has to take medications morning, night, morning, morning, every day, every day, it's that you have to first change his habits, and so that… he doesn't like to do, and so it's difficult (IDI, man of unknown status, 35–49, Bouaké).

ART was also thought to have negative effects on men's health as a result of side effects. In the first few months of ART in particular, men reported that side effects can betray a man's HIV-positive status to his social network or interfere with his ability to work. As one MLHIV in treatment explained, "But the first month on ARVs was not easy at all. I would not vomit but I was exhausted. I felt weak, tired. I could not even do my job" (IDI, MLHIV in treatment, 25–34, Bouaké).

Discussion

Across multiple value domains—sexuality, work and financial success, family, social status, and health—men's perceptions of ART were linked with maintaining or restoring agency or strength. Some men explained how ART, for example, could reduce the risk of transmitting HIV to their partners or their children once they were virally suppressed, thereby restoring sexual agency. ART may also offer the opportunity to regain sexual strength to procreate (and to demonstrate virility) by, for example, reducing concerns about transmitting HIV to future children. ART has the potential to help a man maintain an outward appearance of good health and thereby minimize potential discrimination and job loss. Treatment may impact men's strength to work, but men voiced concerns about how medication regimens and side effects could also limit one's ability to work. ART adherence may give men the ability to fulfill their decision-making and provider roles within the family and household. ART can also allow men to remain economically independent and minimize the risk of social rejection and stigma. Appearance of good health was viewed as mitigating HIV-related stigma and allowed men to maintain respect and "live normally." At the same time, initiation and adherence to ART could also pose challenges to one's social status through unintentional disclosure. Finally, while ART was seen as having the power to improve men's physical and mental health, reduce HIV-related symptoms, or provide men with an appearance of good health, men frequently evoked concerns about long-term, lifelong treatment and side effects that could restrict men's strength and independence. Together, these findings suggest that ART is perceived not only to influence Ivoirian men's health, but in some cases to reduce the threats from HIV to other aspects of their lives that they value as well. Furthermore, while there were contradictions raised about the potential benefits and challenges associated with ART, men described these benefits and challenges within the context of the values highlighted in the MVF (Fig. 1).

This article extends findings from previous research in sub-Saharan Africa identifying barriers men face to getting tested for HIV as well as initiating and adhering to ART if diagnosed positive [2, 8–14]. Studies in other settings [8, 10, 11, 25, 31, 44], as well as recent work in Côte d'Ivoire [33], have highlighted the threat of HIV to domains of masculinity including strength and agency as well as core values such as social status and health that shape men's HIV care-seeking practices. This study builds on prior findings to examine these barriers and the role ART can play, particularly in light

of Test and Treat policies, in mitigating the perceived threat of HIV to men's core values. Findings from this exploratory study may be used to inform future research and interventions focused on HIV testing and engagement in care for men. In particular, future work should examine whether men's perceptions of ART—and how they intersect with core domains of masculinity—influence their HIV-related health-seeking behaviors across the HIV care continuum.

In addition to others' calls for gender transformative approaches to the critical exploration of masculine norms, expectations, and roles [31, 32, 45], there are also opportunities for future SBC approaches. SBC programs could benefit from giving greater attention to men's values and the role of ART in restoring men's strength and agency. This approach marks a departure from a singular focus on health to consider more comprehensively how HIV threatens other aspects of men's lives, both in Côte d'Ivoire and similar settings. SBC programs working at multiple socio-ecological levels, including at the individual level (e.g. men's self-perceptions and self-confidence or self-efficacy), as well as at the couple (e.g. disclosure), household (e.g. concerns about work and family roles), or community level (e.g. HIV-related stigma and social support), can draw on these findings to inform approaches that highlight how ART can address not only men's health, but also concerns they have about their sexuality, work and financial success, family, and social status. Health care workers may also find the MVF [33] and our application of it to ART useful when identifying counseling opportunities or other strategies for use with MLHIV. For example, men's concerns about the threat that ART poses to independence, as a result of treatment fatigue and potential side effects, could be addressed by prioritizing new service delivery models including community ART distribution that reduce the burden on men's time and travel to health facilities. Reframing discussions of ART within the context of men's HIV-related fears may help men re-evaluate cost–benefit assessments regarding HIV testing and treatment and enable them to engage earlier in the HIV care continuum. In particular, SBC programs that are relevant to both MLHIV's lived experiences as well as those of men in the general population can be part of comprehensive HIV strategies. While there were similarities that emerged between MLHIV and men of unknown status in this qualitative study, further studies should explore whether and how their perspectives and lived experiences differ. Specific SBC programs for MLHIV in Côte d'Ivoire should focus on those domains that are of particular relevance to them, while also addressing the domains identified as priorities for the general population of Ivoirian men.

The burden of daily medication regimens and potential side effects associated with ART may continue to pose a challenge for men and impact ongoing adherence and retention in care if the potential benefits to men's agency and strength are not appreciated. Fear of side effects have been documented as barriers to treatment initiation in other areas of Sub-Saharan Africa [4], and fear of side effects led some men in this study—although already diagnosed—to delay treatment initiation as their physical health had not yet been affected. Efforts to address these concerns should remain an important focus of interventions. With new Test and Treat guidelines now in place in Côte d'Ivoire, delaying treatment because one does not feel sick will remain an important issue, particularly for men whose concerns about daily medication or side effects outweigh the perceived benefits of treatment. SBC approaches that address treatment fatigue or side effects, either by highlighting the availability of new treatments with fewer side effects or by emphasizing how early diagnosis can reduce the risk of more severe side effects, could be important avenues for such interventions.

This study was strengthened by its recruitment of men who had been diagnosed with HIV, both those in and not in treatment, as well as those of unknown HIV status. Recruitment of participants who were from different age cohorts as well as from three distinct geographic areas enabled the researchers to interview participants with a variety of lived experiences. The iterative development of the codebook, double-coding of transcripts to establish consistency and consensus, as well as the use of memos throughout data collection ensured analytical rigor during analysis and interpretation.

At the same time, it is essential to consider the limitations of this study. As a qualitative study, the findings are not representative of all experiences of MLHIV or of men's attitudes about HIV or ART in Côte d'Ivoire. For example, these findings may not translate to the experiences and perceptions of men living in rural areas, in places where availability and accessibility of HIV-related care is limited, or in settings with divergent social and gender norms. Instead, the use of IDIs and FGDs was designed to investigate common themes across men's lived experiences and social norms that could inform future studies at the intersection of HIV, ART, and masculinities while informing programmatic recommendations. The collection of data from men identified a range of experiences. Future studies with men's partners and families may further our understanding of men's perceptions of HIV. An exploration of whether the MVF [33] could be relevant to work on HIV with women or other vulnerable populations in Côte d'Ivoire, such as men who have sex with men [46], is also necessary. Finally, the analyses presented here were conducted to explore qualitatively whether and how ART may mitigate the threat of HIV to the core domains of masculine values described in the MVF. Rather than taking a solely inductive approach, these findings are the product of both inductive and deductive analytical processes. Consequently, the results presented here are not necessarily a reflection of all major thematic areas emerging from

the qualitative data gathered and analyzed by the research team. By taking an inductive and deductive approach to data analysis, it is possible that the research team did not capture all possible ways in which ART and masculinity intersect in men's daily lives.

Conclusions

We have described how men's perceptions of ART identify the potential for treatment to alleviate the threat of HIV to masculine value domains. This is of substantial importance in developing SBC approaches to reach men and achieve better testing, linkage to care, and viral suppression outcomes across the treatment continuum. Our exploration of men's knowledge of HIV treatment in conjunction with their perceptions of ART suggests that framing ART as a solution to the threats of HIV against men's values—rather than simply in biomedical terms—is likely to have a greater impact on men's HIV-related healthcare seeking behaviors and ongoing adherence. These findings can be used to develop SBC approaches that highlight the potential for ART to address the perceived threats of HIV to men's core value domains. Further, the findings may strengthen service-side interventions by enabling providers to better counsel men about the issues most relevant to male clients and, in doing so, encourage greater retention in care. Programmatic efforts could be focused on the individual level, such as through the development of individual-level messages about how ART allows men to continue to work and provide for their family, as well as identifying other (e.g. facility, community, structural, or policy-level) avenues to address challenges or barriers to ART uptake and retention. Mutually reinforcing messages can be used across the continuum to build treatment literacy in a way that does not blame, shame, or rouse fear, but instead reframes service-seeking and engagement in care in an empowering way that builds on male clients' strengths and existing values. SBC programs have an essential role to play in supporting men in treatment initiation, helping them appreciate the urgency of immediate enrollment in treatment while still feeling healthy, and ensuring that adherence and viral load are well understood. In understanding male values and perceptions in more depth, a comprehensive package of SBC approaches built on those insights can be most effective in ensuring a client-centered approach and improving male engagement across the HIV continuum.

Acknowledgements The authors would like to acknowledge the men and health workers who so graciously shared their stories and invaluable information with us for this research study. We would also like to thank the data collection team who expertly gathered the narratives of men presented here and provided input during the data analysis process. The research presented here was supported by funding from PEPFAR through the United States Agency for International Development [Cooperative Agreement #AID-OAA-A-12-00058] to the Johns Hopkins Center for Communication Programs. The findings and conclusions in this report are those of the authors and do not necessarily represent the official position of the United States Government, USAID, PEPFAR, or any other affiliate organizations or institutions.

Compliance with Ethical Standards

Conflict of interest Two of the authors, Seifert Ahanda and Thaddeus, were employed by the funding agency at the time of data collection.

Ethical Approval All procedures performed in studies involving human participants were in accordance with the ethical standards of the institutional and/or national research committee and with the 1964 Helsinki declaration and its later amendments or comparable ethical standards.

Informed Consent Informed consent was obtained from all individual participants included in the study.

References

1. UNAIDS. 90-90-90: an ambitious treatment target to help end the AIDS epidemic. Geneva, Switzerland: Joint United Nations Programme on HIV/AIDS (UNAIDS), 2014 Contract No.: JC2684; 2014.
2. WHO. Progress report 2016: prevent HIV, test and treat all. WHO support for country impact. WHO/HIV/2016.24. Geneva, Switzerland: WHO; 2016.
3. Shah M, Risher K, Berry SA, Dowdy DW. The epidemiologic and economic impact of improving HIV testing, linkage, and retention in care in the United States. Clin Infect Dis. 2015;62(2):220–9.
4. Adams AK, Zamberia AM. "I will take ARVs once my body deteriorates": an analysis of Swazi men's perceptions and acceptability of Test and Start. Afr J AIDS Res. 2017;16(4):295–303.
5. WHO. Treat all: policy adoption and implementation status in countries. WHO/HIV/2017.58. Geneva, Switzerland: WHO; 2017.
6. UNAIDS. Country factsheets. Côte d'Ivoire. 2016. http://www.unaids.org/en/regionscountries/countries/ctedivoire. Accessed 5 May 2018.
7. Maheu-Giroux M, Vesga JF, Diabaté S, Alary M, Baral S, Diouf D, et al. Population-level impact of an accelerated HIV response plan to reach the UNAIDS 90-90-90 target in Côte d'Ivoire: insights from mathematical modeling. PLoS Med. 2017;14(6):e1002321.
8. Siu GE, Wight D, Seeley J. How a masculine work ethic and economic circumstances affect uptake of HIV treatment: experiences of men from an artisanal gold mining community in rural eastern Uganda. J Int AIDS Soc. 2012;15(Suppl 1):1–9.
9. Nyamhanga TM, Muhondwa EPY, Shayo R. Masculine attitudes of superiority deter men from accessing antiretroviral therapy in Dar es Salaam, Tanzania. Glob Health Action. 2013;6(1):21812.
10. Chikovore J, Gillespie N, McGrath N, Orne-Gliemann J, Zuma T, Group A 12249 TS. Men, masculinity, and engagement with treatment as prevention in KwaZulu-Natal, South Africa. AIDS Care. 2016;28(sup3):74–82.
11. Shand T, Thomson-de Boor H, van den Berg W, Peacock D, Pascoe L. The HIV blind spot: men and HIV testing, treatment and care in sub-Saharan Africa. IDS Bull. 2014;45(1):53–60.
12. Parrott FR, Mwafulirwa C, Ngwira B, Nkhwazi S, Floyd S, Houben RMGJ, et al. Combining qualitative and quantitative evidence to determine factors leading to late presentation for antiretroviral therapy in Malawi. PLoS ONE. 2011;6(11):e27917.

13. Gari S, Martin-Hilber A, Malungo JRS, Musheke M, Merten S. Sex differentials in the uptake of antiretroviral treatment in Zambia. AIDS Care. 2014;26(10):1258–62.
14. Druyts E, Dybul M, Kanters S, Nachega J, Birungi J, Ford N, et al. Male sex and the risk of mortality among individuals enrolled in antiretroviral therapy programs in Africa: a systematic review and meta-analysis. AIDS. 2013;27(3):417–25.
15. Kagee A, Remien RH, Berkman A, Hoffman S, Campos L, Swartz L. Structural barriers to ART adherence in Southern Africa: challenges and potential ways forward. Glob Public Health. 2011;6(1):83–97.
16. Cornell M, Schomaker M, Garone DB, Giddy J, Hoffmann CJ, Lessells R, et al. Gender differences in survival among adult patients starting antiretroviral therapy in South Africa: a multicentre cohort study. PLoS Med. 2012;9(9):e1001304.
17. Dovel K, Yeatman S, Watkins S, Poulin M. Men's heightened risk of AIDS-related death: the legacy of gendered HIV testing and treatment strategies. AIDS. 2015;29(10):1123–5.
18. Hodgson I, Plummer ML, Konopka SN, Colvin CJ, Jonas E, Albertini J, et al. A systematic review of individual and contextual factors affecting ART initiation, adherence, and retention for HIV-infected pregnant and postpartum women. PLoS ONE. 2014;9(11):e111421.
19. Holtzman CW, Brady KA, Yehia BR. Retention in care and medication adherence: current challenges to antiretroviral therapy success. Drugs. 2015;75(5):445–54.
20. Hoffmann CJ, Mabuto T, McCarthy K, Maulsby C, Holtgrave DR. A framework to inform strategies to improve the HIV care continuum in low-and middle-income countries. AIDS Educ Prev. 2016;28(4):351–64.
21. Govindasamy D, van Schaik N, Kranzer K, Wood R, Mathews C, Bekker L-G. Linkage to HIV care from a mobile testing unit in South Africa by different CD4 count strata. J Acquir Immune Defic Syndr. 2011;58(3):344–52.
22. Johnson LF. Access to antiretroviral treatment in South Africa, 2004–2011. South Afr J HIV Med. 2012;13(1):22–7.
23. Kranzer K, Zeinecker J, Ginsberg P, Orrell C, Kalawe NN, Lawn SD, et al. Linkage to HIV care and antiretroviral therapy in Cape Town, South Africa. PLoS ONE. 2010;5(11):e13801.
24. Musheke M, Ntalasha H, Gari S, McKenzie O, Bond V, Martin-Hilber A, et al. A systematic review of qualitative findings on factors enabling and deterring uptake of HIV testing in sub-Saharan Africa. BMC Public Health. 2013;13(220):1–16.
25. Siu GE, Seeley J, Wight D. Dividuality, masculine respectability and reputation: how masculinity affects men's uptake of HIV treatment in rural eastern Uganda. Soc Sci Med. 2013;89:45–52.
26. Camlin CS, Ssemmondo E, Chamie G, El Ayadi AM, Kwarisiima D, Sang N, et al. Men "missing" from population-based HIV testing: insights from qualitative research. AIDS Care. 2016;28(sup3):67–73.
27. Merten S, Kenter E, McKenzie O, Musheke M, Ntalasha H, Martin-Hilber A. Patient-reported barriers and drivers of adherence to antiretrovirals in sub-Saharan Africa: a meta-ethnography. Trop Med Int Health. 2010;15(s1):16–33.
28. Gottert A, Barrington C, McNaughton-Reyes HL, Maman S, MacPhail C, Lippman SA, et al. Gender norms, gender role conflict/stress and HIV risk behaviors among men in Mpumalanga, South Africa. AIDS Behav. 2017;22(6):1858–69.
29. Morrell R, Jewkes RK, Lindegger G, Hamlall V. Hegemonic masculinity: reviewing the gendered analysis of men's power in South Africa. S Afr Rev Sociol. 2013;44(1):3–21.
30. Hanass-Hancock J, Myezwa H, Nixon SA, Gibbs A. "When I was no longer able to see and walk, that is when I was affected most": experiences of disability in people living with HIV in South Africa. Disabil Rehabil. 2015;37(22):2051–60.
31. Mburu G, Ram M, Siu G, Bitira D, Skovdal M, Holland P. Intersectionality of HIV stigma and masculinity in eastern Uganda: implications for involving men in HIV programmes. BMC Public Health. 2014;14(1):1061.
32. Sikweyiya YM, Jewkes R, Dunkle K. Impact of HIV on and the constructions of masculinities among HIV-positive men in South Africa: implications for secondary prevention programs. Glob Health Action. 2014;7(1):1–13.
33. Naugle DA, Tibbels N, Hendrickson ZM, Dosso A, Van Lith L, Mallalieu EC, et al. Bringing fear into focus: the intersections of HIV and masculine gender norms in Côte d'Ivoire. 2019.
34. DHS-MICS. Extrait des données EDS. Prévalence du VIH en cote d'Ivoire: Résultat de l'EDS-MICS 2011–2012. Calverton, MD, USA: MEASURE DHS, ICF International; 2012. https://www.dhsprogram.com/pubs/pdf/HF46/HF46.pdf.
35. Health Communication Capacity Collaborative. The facilitator guide: Brothers for Life Plus Community Groups. Baltimore, MD: Johns Hopkins Center for Communication Programs; 2017. https://healthcommcapacity.org/hc3resources/facilitator-guide-brothers-life-plus-community-groups/. Accessed 8 Apr 2018.
36. RGPH. Rapport d'exécution et présentation des principaux résultats. Recensement Général de la Population et de l'Habitat; 2014. http://www.ins.ci/n/documents/RGPH2014_expo_dg.pdf.
37. The Editors of Encyclopaedia Britannica. Bouaké. In: Encyclopaedia Britannica. Encyclopaedia Britannica, Inc.; 2013. p. 1. https://www.britannica.com/place/Bouake.
38. The Editors of Encyclopaedia Britannica. San-Pédro. In: Encyclopaedia Britannica. Encyclopaedia Britannica, Inc.; 2010. p. 1. https://www.britannica.com/place/San-Pedro-Cote-dIvoire.
39. The Editors of Encyclopaedia Britannica. Abidjan. In: Encyclopaedia Britannica. Encyclopaedia Britannica, Inc.: 2015. p. 1.
40. The World Bank. Côte d'Ivoire: overview. 2018. http://www.worldbank.org/en/country/cotedivoire/overview. Accessed 9 Aug 2018.
41. Government of Côte d'Ivoire. Côte d'Ivoire: Histoire. 2018. http://www.gouv.ci/_histoire.php. Accessed 9 Aug 2018.
42. Goverment of Côte d'Ivoire. Côte d'Ivoire: La Fiche Signalétique de la Côte d'Ivoire. 2018. http://www.gouv.ci/_fiche-signaletique.php. Accessed 9 Aug 2018.
43. ATLAS/ti. ATLAS/ti. Version 1.0.51. (Computer sofware for Mac). Scientific Sofware Development, Berlin; 2015.
44. Nyamhanga TM, Muhondwa EP, Shayo R. Masculine attitudes of superiority deter men from accessing antiretroviral therapy in Dar es Salaam. Tanzania. Glob Heal Action. 2013;6:21812.
45. Fleming PJ, Barrington C, Powell W, Gottert A, Lerebours L, Donastorg Y, et al. The association between men's concern about demonstrating masculine characteristics and their sexual risk behaviors: findings from the dominican republic. Arch Sex Behav. 2018;47(2):507–15.
46. Aho J, Hakim A, Vuylsteke B, Semde G, Gbais HG, Diarrassouba M, et al. Exploring risk behaviors and vulnerability for HIV among men who have sex with men in Abidjan, Cote d' Ivoire: poor knowledge, homophobia and sexual violence. PLoS ONE. 2014;9(6):e99591.

Publisher's Note Springer Nature remains neutral with regard to jurisdictional claims in published maps and institutional affiliations.
